# Confronting the Paradox of Enrichment to the Metacommunity Perspective

**DOI:** 10.1371/journal.pone.0082969

**Published:** 2013-12-16

**Authors:** Céline Hauzy, Grégoire Nadin, Elsa Canard, Isabelle Gounand, Nicolas Mouquet, Bo Ebenman

**Affiliations:** 1 Department of Physics, Chemistry and Biology, Linköping University, Linköping, Sweden; 2 Université Pierre et Marie Curie, UMR7625 - Ecologie et Evolution, Paris, France; 3 Institut National de la Recherche Agronomique, USC2031 - Ecologie des Populations et Communautés, Paris, France; 4 CNRS, UMR7598 - Laboratoire Jacques-Louis Lions, Paris, France; 5 Institut des Sciences de l′Evolution, Université de Montpellier II, Montpellier, France; Institut Maurice-Lamontagne, Canada

## Abstract

Resource enrichment can potentially destabilize predator-prey dynamics. This phenomenon historically referred as the "paradox of enrichment" has mostly been explored in spatially homogenous environments. However, many predator-prey communities exchange organisms within spatially heterogeneous networks called metacommunities. This heterogeneity can result from uneven distribution of resources among communities and thus can lead to the spreading of local enrichment within metacommunities. Here, we adapted the original Rosenzweig-MacArthur predator-prey model, built to study the paradox of enrichment, to investigate the effect of regional enrichment and of its spatial distribution on predator-prey dynamics in metacommunities. We found that the potential for destabilization was depending on the connectivity among communities and the spatial distribution of enrichment. In one hand, we found that at low dispersal regional enrichment led to the destabilization of predator-prey dynamics. This destabilizing effect was more pronounced when the enrichment was uneven among communities. In the other hand, we found that high dispersal could stabilize the predator-prey dynamics when the enrichment was spatially heterogeneous. Our results illustrate that the destabilizing effect of enrichment can be dampened when the spatial scale of resource enrichment is lower than that of organismss movements (heterogeneous enrichment). From a conservation perspective, our results illustrate that spatial heterogeneity could decrease the regional extinction risk of species involved in specialized trophic interactions. From the perspective of biological control, our results show that the heterogeneous distribution of pest resource could favor or dampen outbreaks of pests and of their natural enemies, depending on the spatial scale of heterogeneity.

## Introduction

Human activities, and especially agriculture, lead to nutrient enrichment of aquatic and terrestrial ecosystems [1], [2], which may change resource availability and alter the dynamic of populations and their persistence [3]–[5]. Rosenzweig [5] showed, on the theoretical ground, that constant resource enrichment for the prey can destabilize population dynamics in predator-prey models. At low resource level, prey and predator dynamics reach a stable equilibrium that becomes unstable when the resource level increases above a threshold value. With increasing enrichment the amplitude of densities fluctuations increases and populations become more prone to extinction. This destabilizing effect of enrichment was called the “paradox of enrichment” by Rosenzweig [5]. Rip and McCann [3] provided recently a new interpretation of this phenomenon and suggested that the paradox of enrichment is a special case of a more general theoretical result that they called the “principle of energy flux”. They showed that the increase in the energy flux between a prey and its predator (relative to the predator mortality rate), which can be caused by an enrichment of prey resources or other factors, destabilizes predator-prey dynamics. Such destabilizing effect of enrichment has been observed in microcosm experiments only, using ciliates and bacteria [6], [7], algae and rotifer [8] or mites [9].

Some observations in natural conditions are consistent with the paradox of enrichment. For instance, in some lakes, the increase in phytoplankton variability has been related to nutrient enrichment [10]. However, such observations remain rare, suggesting that the initial simple models are missing important factors or mechanisms that counteract the destabilizing effect of enrichment in most communities. The proposed mechanisms include (for review see [4]) the presence of several prey with various accessibility to the predator [11]–[13], interference between predators (functional response with predator dependence [14], [15], density dependence in predator mortality [16]), prey defenses induced by the predator [17], movements in spatially structured communities [18] and the presence of spatial refuges for the prey [19]. These two last mechanisms have underlined the importance of spatial dynamics and spatial heterogeneity for the paradox of enrichment, but their importance is still poorly understood.

Many communities exchange organisms within spatially structured networks called metacommunities [20]–[22]. Movements of organisms between communities vary from frequent to rare. Among the numerous examples of such metacommunities, we can cite phytophagous mites and their predators which disperse between the patches made of the apple trees of orchards [23]. Moreover, the environmental conditions in the various parts of the metacommunities can be different (spatial heterogeneity). For instance, lakes are structured by a vertical gradient of light from the surface to deep water which shapes primary producer distribution in the water column [24] and zooplankton and their predator (fish) move frequently between the pelagic part and the benthic part beneath [25]. In agricultural landscapes, phytophagous arthropods and their natural enemies move between crop patches and semi-natural grassland patches [26]. The metacommunity theory provides a relevant framework to study the importance of dispersal and of spatial heterogeneity on population dynamics in such spatially structured systems [21], [22], [27].

The stabilizing effect of dispersal on prey and predator persistence has been observed in laboratory experiments [28]–[33], in field experiments [23] and in natural communities [34]. Metacommunity theory has shown that dispersal can stabilize prey and predator dynamics at the local and at the regional scales via three mechanisms [27], [35]. First, low or intermediate dispersal rates can promote the stability of local population dynamics when dispersal from asynchronous populations leads to negative density-dependence in per capita growth rate [27], [36]. Second, high dispersal rates in heterogeneous landscapes can lead to non-linear averaging of the demographic parameters which may stabilize predator-prey dynamics [37]. Third, intermediate dispersal rates can also dampen the amplitude of regional density fluctuations when population dynamics are spatially asynchronous [18], [38] (called "statistical stabilization" [27], [38]). Jansen [18] studied explicitly the paradox of enrichment in uniform landscapes. He showed that this statistical stabilization mechanism could dampen the destabilizing effect of an equal enrichment in all communities. This result has underlined the importance of spatial dynamics for the paradox of enrichment, but was restricted to uniform landscapes.

It is likely that natural metacommunities occupy heterogeneous landscapes [39], [40]. For instance, resource availability or the strength of predator-prey interaction can vary from patch to patch. The presence of spatial refuges for the prey, where the predation risk is decreased, can dampen the destabilizing effect of enrichment on the dynamics of prey and predators [19]. Whereas spatial heterogeneity of resource availability exists in absence of human disturbance, human activities can also increase differences between patches. For instance, lake eutrophication increases phytoplankton biomass in upper layers, which reduces light availability for benthic algae growing in deeper layers of the water column [41]. In terrestrial landscapes, the conversion of semi-natural meadows into crop patches can increase the resource availability for herbivorous insects and the spatial heterogeneity of this resource. However, the effect of enrichment distribution over the landscape on the dynamics of prey and their predators in metacommunities remains poorly studied.

Here we studied the combined effects of dispersal and of enrichment distribution over the landscape on the stability of prey and predator dynamics in metacommunities. We adapted the original Rosenzweig-MacArthur predator-prey model to the metacommunity framework, by considering population dynamics within patches, dispersal of organisms between patches and varying carrying capacity of the prey from patch to patch (spatial heterogeneity). We investigated how the effect of enrichment distribution interacts with the effect of dispersal to modify the stability of population dynamics. Our results show that for a given regional enrichment, spatial heterogeneity in resource distribution and dispersal can shift the destabilizing effect of enrichment to higher values, thus potentially buffering the paradox of enrichment.

## Methods

### The model

Our general model describes the dynamics of a prey and its predator occupying a landscape of *M* patches, which differ by resource availability for the prey. The local dynamics of the prey and of the predator follow a Rosenzweig-MacArthur model [42]. In the absence of the predator, the prey growth is logistic and prey density is limited by the carrying capacity *K_i_*. We assume that increased resource availability for the prey in patch *i* increases the carrying capacity *K_i_*, but does not affect the intrinsic growth rate *r* of the prey. The predator consumption of the prey follows a Holling type II functional response [43] with an attack rate *a* and a handling time *t*_h_. The predator converts a proportion *e* of consumed prey density into its own density. The mortality rate of the predator *m* is constant. Following these assumptions, an isolated community has one equilibrium where the prey and the predator can coexist if the carrying capacity in the patch is sufficiently high to allow the predator consumption to compensate its own mortality (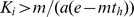). In other words, the predator can maintain positive density if its efficiency is sufficiently high (sufficiently high attack rate *a* or conversion efficiency *e* and sufficiently low mortality rate *m* or handling time *t*_h_). When the predator can maintain, this equilibrium is stable if and only if the carrying capacity *K*_i_ in the isolated patch is lower than 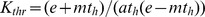 (Appendix S1). This stability threshold is specific to a given predator and decreases with predator efficiency.

The dispersal rates of the prey and of the predator, whose relative values can vary within a large range [44], follow the rules: (1) the prey and the predator disperse from one patch to another at constant rates (passive dispersal) denoted *d_N_* and *d_P_* respectively; (2) the dispersal rates *d_N_* and *d_P_* are the same for all patch pairs (global dispersal). Following all these assumptions, the variations of the prey and of the predator densities, *N* and *P*, over time in the patch *i* are given by the equations: 
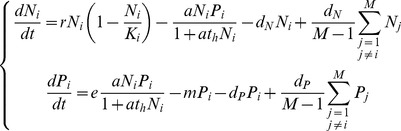
(1)


The local enrichment of patch *i* in prey resource will be represented by an increase *E*_i_ of the carrying capacity *K*_i_. The regional enrichment *E* is defined as the sum of the local enrichments in the *M* patches: 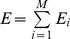. We control the spatial distribution of enrichment by varying the distribution of the *E*_i_'s. The spatial distribution of enrichment is described by *α* which ranges between two extreme situations: from a uniform distribution (*α* = 0), for which the enrichment is evenly distributed over patches, to the maximal level of heterogeneity (*α* = 1), when the enrichment is concentrated in a single patch. For a two-patch landscape (*M* = 2), the spatial distribution of enrichment is computed as 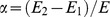. For the sake of simplicity, we consider that carrying capacities *K*_i_ are identical in all patches before enrichment and that they are equal to *K*_0_. Hence heterogeneous distribution of enrichment leads to heterogeneous distribution of carrying capacities.

### Analyses

We investigated the effect of regional enrichment *E*, of its spatial distribution α and of dispersal rates *d_N_* and *d_P_* on the stability of the long-term dynamics of the metacommunity. In the limiting cases where dispersal is zero or where dispersal is infinitely high, we could perform an analytical study of the dynamics for metacommunities made of *M* patches. In order to study the dynamics for dispersal rates between these two limiting cases, we carried out numerical simulations and, for the sake of simplicity, we restricted the analysis to two-patch metacommunities (*M* = 2). Moreover, to test the robustness of our analytical results that assume equal dispersal rates for the prey and the predator, we explored cases with contrasting dispersal rates of the prey and of the predator (*d*_P_ = 100 *d_N_* and *d*_P_ = 0.01 *d_N_*).

The stability of the dynamics of the metacommunity was investigated using two methods. First, we studied the stability in the neighborhood of the equilibrium of the metacommunity. Equilibrium densities were derived analytically when possible or computed numerically using the function fsolve in Matlab 7.6 (Levenberg-Marquardt algorithm). We retained equilibriums with positive densities. The stability was assessed checking the eigenvalues of the Jacobian matrix of the system (1). When the real parts of all eigenvalues are negative, the equilibrium is stable; when at least one eigenvalue has a positive real part, the equilibrium is unstable. This measurement of the stability of population dynamics gives binary information: it indicates whether the population dynamics converge to the equilibrium or not.

To complete this information, we used a second measurement to characterize the stability of population dynamics that do not reach equilibrium. We simulated the population dynamics in the landscape by numerically integrating equations (1) over a period of 30000 time units using the function ode15s in Matlab 7.6 (adaptive step size and variable order integrator based on the numerical differentiation formulas). According to the simulations, asymptotic regime (stable equilibrium or a stable limit cycle) was reached after 20000 units of time and we performed measurements on time series between 20000 and 30000 units of time. The stability of the population dynamics was quantified by the amplitude of the fluctuations of population densities over time and by the minimal densities reached in the time series. When the amplitude was equal to zero, we checked that the densities reached at the asymptotic regime were the same as the equilibrium densities found using numerical computation (see above). We considered that the stability of the population dynamics was lower when the amplitude was higher. The population extinction risk was considered to be higher for lower minimal densities.

In our numerical simulations, we used several random initial densities to detect potential alternative stable steady states that can be observed in predator-prey metacommunity models with identical patches [18], [45]. We chose parameters to allow the persistence of the predator in an isolated patch (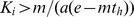). We explored the stability of the population densities in two-patch metacommunities for a wide range of parameter values: growth rate of the prey *r* = [1], [10], conversion efficiency *e* = [0.1,1], attack rate *a* = [1], [10], handling time *t_h_* = [0.01,0.1] and mortality rate of the predator *m* = [0.1,1].

## Results

### (1) Effect of spatial heterogeneity on metacommunity stability in two extreme scenarios

#### (a) No dispersal

First, we studied analytically the effect of the spatial distribution of enrichment on the metacommunity equilibrium in the trivial limiting case where there is no dispersal, i.e. when the metacommunity is simply a set of M isolated communities. Without dispersal, the metacommunity equilibrium is stable if the equilibriums of each of the M isolated communities are stable. If the carrying capacity of at least one community crosses the stability threshold of an isolated patch (K_thr_), then the metacommunity equilibrium is unstable.

**Figure 1:**
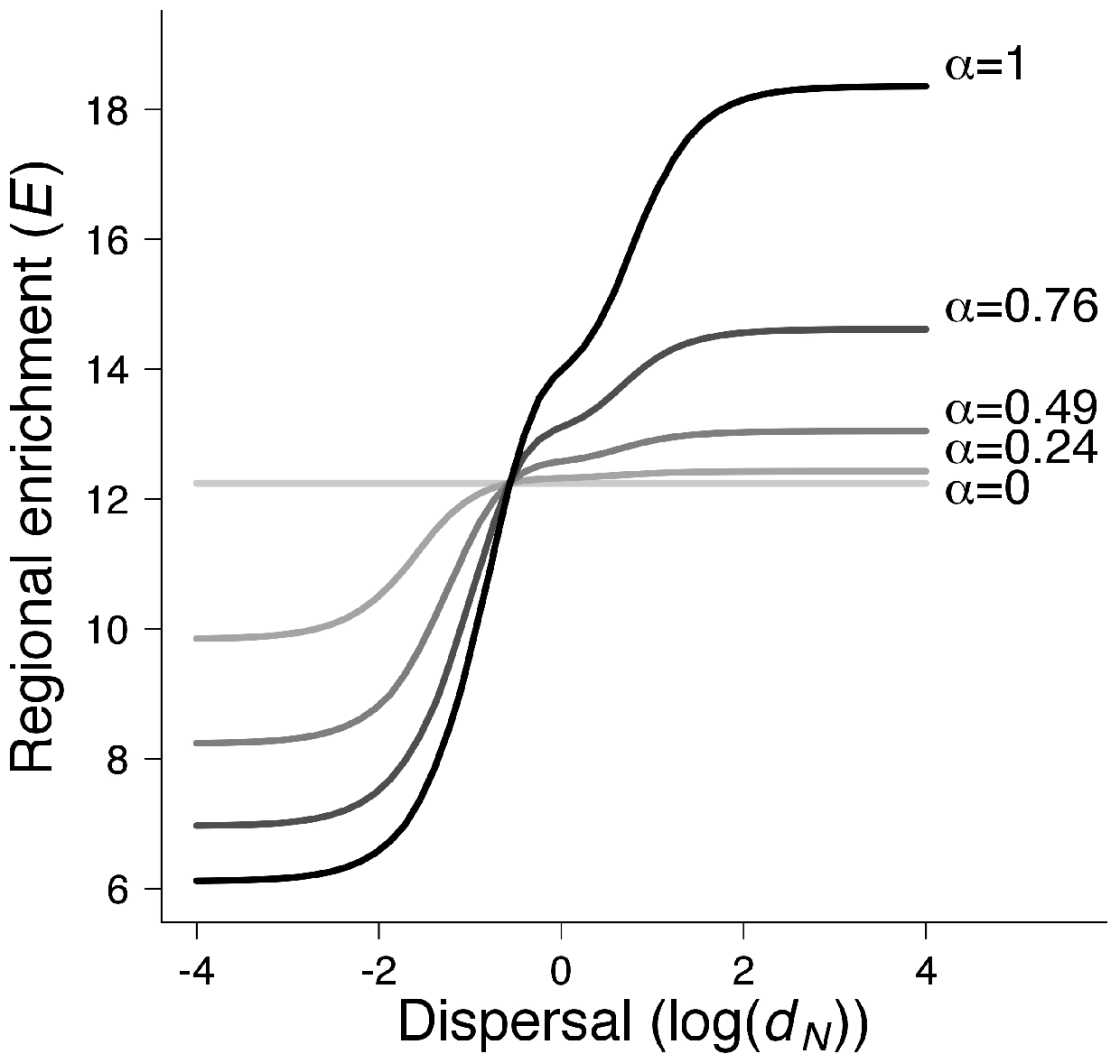
Effect of regional enrichment (*E*), of its spatial distribution (α) and of dispersal rate (*d_N_* = *d_P_*) on metacommunity stability. Metacommunities have two patches (*M* = 2). When enrichment is zero, the carrying capacities are equal to *K*_0_ and they are below the stability threshold of an isolated patch. The stability of metacommunity equilibrium is determined using the sign of the real part of eigenvalues of the Jacobian matrix. For each α value, the equilibrium is stable below the solid line (the real part of each eigenvalue is negative) and it is unstable above the solid line (at least one eigenvalue has a positive real part). The solid lines then represent the enrichment threshold, 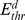, for which the metacommunity is destabilized. Parameters values: *r* = 10, *e* = 0.1, *a* = 5, *t_h_*  = 0.01, *m* = 1, *K*_0_ = 18.3.

The effect of the spatial distribution of enrichment when dispersal is null can be derived from this property. Consider a metacommunity of *M* patches where the carrying capacity is the same in every patch (*K*_0_) and is below the threshold *K*_thr_. We found that the metacommunity equilibrium is destabilized at lower level of regional enrichment when enrichment is concentrated in one patch (α = 1), than when it is evenly distributed (α = 0) (Appendix S1). Figure 1 provides an illustration of this effect of enrichment distribution on the metacommunity stability for very low dispersal rates (log(*d*_N_) = −4)).

#### (b) Infinite dispersal

Second, we studied analytically how the stability of the metacommunity equilibrium depends on the spatial distribution of enrichment in the limiting case where dispersal is infinitely high. In that case, we found a mathematical approximation of the metacommunity (Appendix S1). The metacommunity follow the dynamic of a Rosenzweig-MacArthur model, where the regional carrying capacity corresponds to the harmonic mean of the local carrying capacities K_i_'s: 
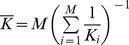
 (Appendix S1).

The regional carrying capacity 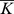 depends on the spatial distribution of enrichment. Again, let's consider a metacommunity where every patch has initially the same carrying capacity (*K*_0_) that is below the stability threshold of an isolated patch (*K*_thr_). When the enrichment is uniform (α = 0), the carrying capacities after enrichment are the same in all patches, and the harmonic mean 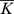 of carrying capacities is equal to their arithmetic mean. When spatial heterogeneity in enrichment distribution increases (α>0), the carrying capacities in some patches become higher than in other patches, and the harmonic mean 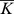 of carrying capacities is lower than the arithmetic mean of the carrying capacities. As a consequence, we found that the metacommunity equilibrium is destabilized at higher level of enrichment (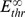) when enrichment heterogeneity is maximal (α = 1) than when enrichment distribution is uniform (α = 0) (Appendix S1). Thus, when dispersal is very high, the metacommunity equilibrium is more robust to enrichment destabilization when the distribution of enrichment is heterogeneous (α>0) than when it is uniform (α = 0). Figure 1 shows an illustration of this positive effect of enrichment distribution on equilibrium stability for high dispersal rates (log(*d*_N_) = 4). This stabilizing effect of enrichment heterogeneity increases when the number of patches in the landscape increases (Appendix S1).

To synthetize, the effect of spatial heterogeneity of enrichment at infinite dispersal is stabilizing whereas it is destabilizing when there is no dispersal (see above).

### (2) Effect of dispersal on metacommunity stability

#### (a) Dynamical stability

**Figure 2:**
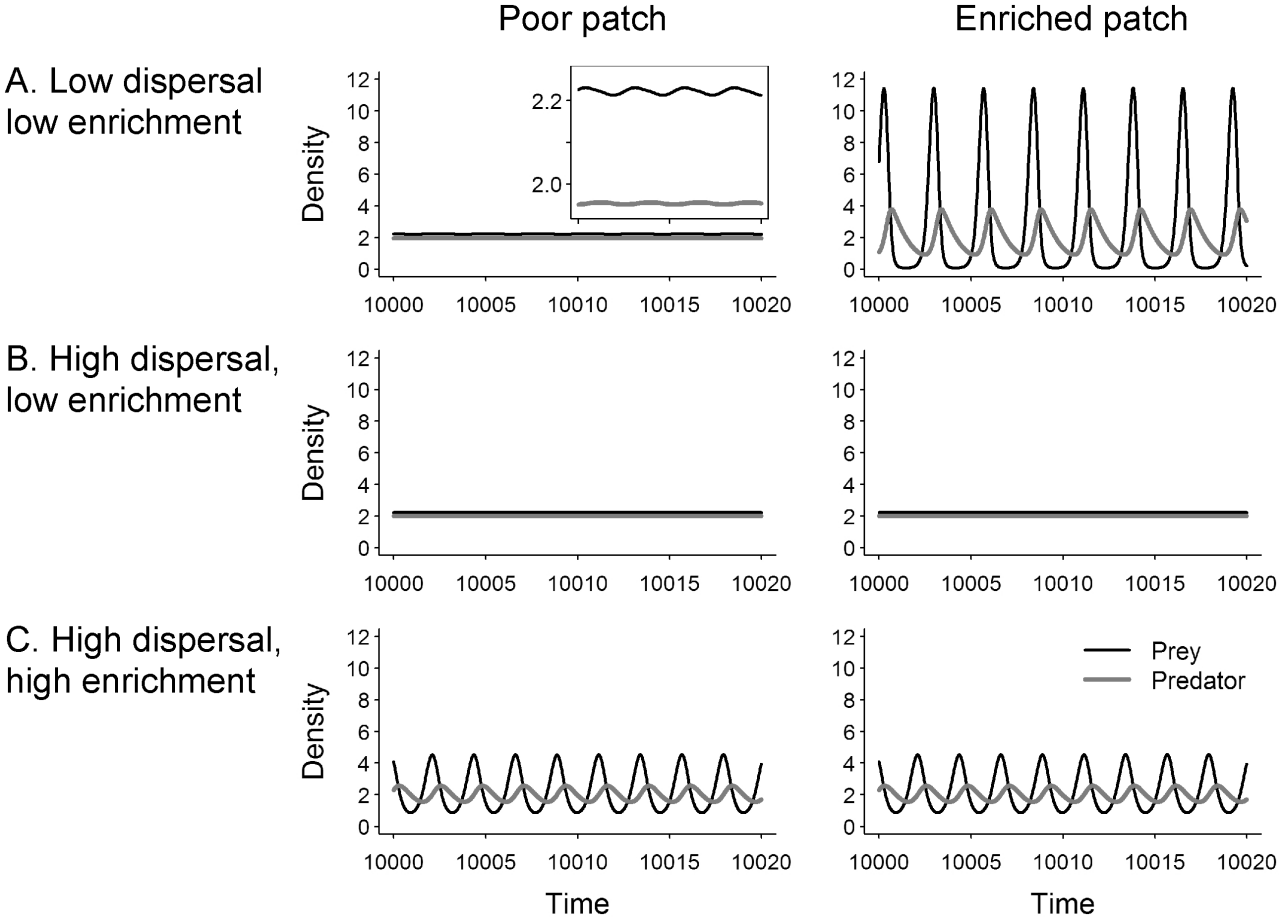
Examples of population dynamics of the prey (black) and of the predator (grey) when spatial heterogeneity is maximal (α = 1). Metacommunities have two patches (*M* = 2), one poor patch (left column), where prey carrying capacity (*K_1_*) is kept constant, and one enriched patch (right column), where prey carrying capacity (*K_2_* =  *K_1_*+*E*) is increased. A. Low dispersal (*d_N_* = 0.001) and low enrichment (*K_2_* = 30). B. High dispersal (*d_N_* = 1000) and low enrichment (*K_2_* = 30). C. High dispersal (*d_N_* = 1000) and high enrichment (*K_2_* = 40). These three cases correspond to the three points labeled respectively A, B and C on the panels of the Figure 3. Other parameters values: *r* = 10, *K_1_* = 18.3, *e* = 0.1, *a* = 5, *t_h_* = 0.01, *m* = 1, *d_P_* =  *d_N_*.

Third, we used numerical computation to study the stability threshold for intermediate dispersal rates and for varying the spatial distribution of enrichment in a two-patch metacommunity (M = 2). Before the regional enrichment of the metacommunity, the carrying capacities in all patches are fixed at a value K_0_. This value is below the stability threshold of an isolated patch and above the carrying capacity for which the metacommunity equilibrium remains stable for any given regional enrichment at very high dispersal rates (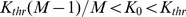). We found that the metacommunity equilibrium goes from stable to unstable when regional enrichment increases and crosses the threshold 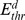 (Plotted in Fig. 1). When the spatial distribution of enrichment is uniform (α = 0), dispersal does not affect the stability of metacommunity equilibrium (Fig. 1). This is consistent with our analytical results obtained for the two previous limiting cases, zero and infinite dispersal (Appendix S1),. By contrast, when enrichment distribution is heterogeneous (α>0), dispersal increases the stability of metacommunity equilibrium (Fig. 1, Fig. S2). For a moderate regional enrichment (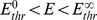), dispersal stabilizes population dynamics (Fig. 2A, B). We note that this relationship between dispersal and stability can be non-monotonic when predator dispersal rate is much higher than prey dispersal rate (Fig. S3, Fig. S4). Moreover, when the spatial heterogeneity in enrichment distribution α increases, the stabilizing effect of dispersal is increased (higher regional enrichment threshold 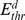) (Fig. 1). These results are consistent with our analytical results: when spatial heterogeneity in enrichment distribution is maximal (α = 1), we found that for any patch number, the enrichment level that destabilizes the equilibrium is lower at low dispersal rates than at infinite dispersal rates (Appendix S1).

Thus, as long as dispersal is sufficiently high, enrichment distribution is heterogeneous and regional enrichment is moderate, the metacommunity equilibrium can be stable even if the carrying capacity in one patch is higher than the stability threshold of an isolated patch (Fig. 1). In this case, population dynamics of the prey and of the predator reach a stable equilibrium in both patches (Fig. 2B).

#### (b)Population variability

Fourth, we investigated the effect of dispersal on population dynamics when the metacommunity equilibrium is unstable. We studied a two-patch metacommunity (M = 2) where the spatial heterogeneity of enrichment distribution is maximal (α = 1), i.e. where the carrying capacity is increased in the "enriched patch" only. The carrying capacity in the "poor patch" is fixed at a value K_0_ below the threshold carrying capacity that destabilizes an isolated patch (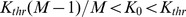).

**Figure 3:**
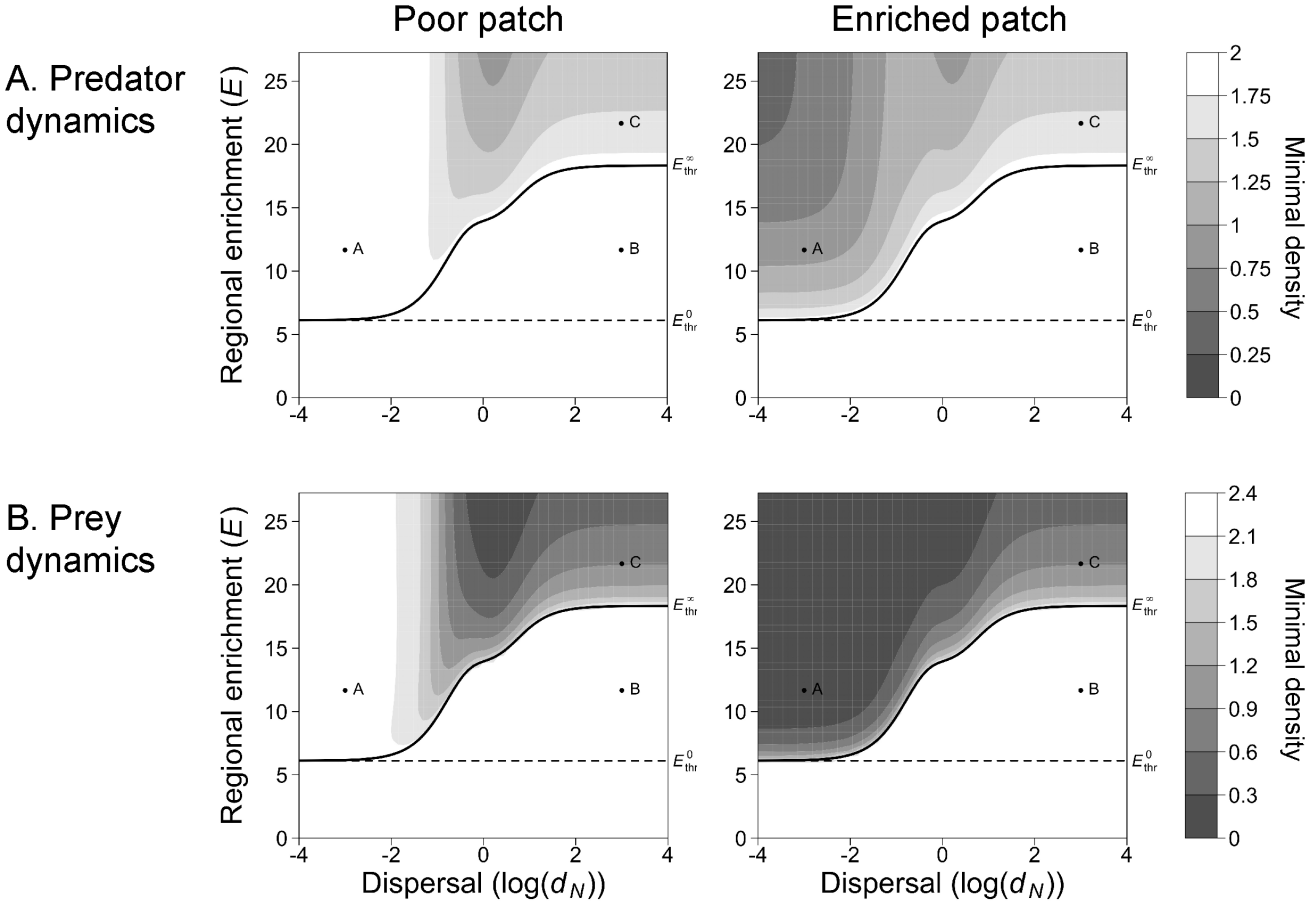
Effect of regional enrichment (*E*) and of dispersal rate (*d_N_* = *d_P_*) on the extinction risk when spatial heterogeneity is maximal (α = 1). When enrichment is zero, the carrying capacities are equal to *K*_0_ and they are below the stability threshold of an isolated patch. Regional enrichment (*E*) increases the carrying capacity in the enriched patch (*K_0_*+*E)*, whereas the carrying capacity in the poor patch is kept constant (*K_0_*). The equilibrium of the two-patch metacommunity (*M* = 2) is stable below the black line. The minimal densities of the predator (A) and of the prey (B) in the poor patch (left column) and in the enriched patch (right column) are represented with grey levels. The higher the minimal density, the lower the extinction risk. At the three points labeled A, B and C, the population dynamics are illustrated in Figure 2. The solid lines then represent the enrichment threshold, 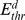, for which the metacommunity is destabilized. 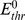 and 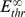 denote the regional enrichment thresholds found analytically respectively for an isolated patch (*d_N_* = *d_P_* = 0) and for the well-mixed metacommunity (*d_N_* = *d_P_* = +∞). Parameters values: *r* = 10, *K_0_* = 18.3, *e* = 0.1, *a* = 5, *t_h_*  = 0.01, *m* = 1.

At very low dispersal rates, population dynamics of the prey and of the predator are similar to the population dynamics in two isolated patches (Fig. 3, S1). The threshold value of regional enrichment 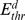 (black line, Fig. 3, S1) tends to the threshold value of an isolated patch 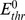
regardless of differences between prey and predator dispersal rates (Fig. S3 and S4). When regional enrichment is higher than this threshold, the densities of the prey and of the predator fluctuate over time in the enriched patch (Fig. 2A). Regional enrichment increases the amplitude of fluctuation of prey and predator densities (Fig. S1) and decreases the minimal densities reached (Fig. 3, above the solid line). By contrast, the population densities in the poor patch remain almost stable: the amplitude of fluctuations is very small (Fig. S1) and minimal densities are slightly decreased (Fig. 3, Fig. 2A). Thus at low dispersal rates, regional enrichment with heterogeneous distribution destabilizes the population dynamics in the patch where enrichment is concentrated without affecting the other patch dynamics. This result is not affected by differences between prey and predator dispersal rates (Fig. S3, Fig. S4).

When dispersal increases, destabilization occurs at higher regional enrichment, hence the metacommunity is more stable (Fig. 3, Fig. S1). In the poor patch, increasing dispersal rates leads to non-monotonic increase in the amplitude of fluctuations of prey and predator densities (Fig. S1) and to non-monotonic decrease in the minimal densities reached (Fig. 3, above the solid line). Conversely, dispersal rates decrease non-monotonically the amplitude of population dynamics (Fig. S1) and increase non-monotonically the minimal densities (Fig. 3, above the solid line) in the enriched patch. These non-monotonic relationships are the consequences of a higher destabilization of predator-prey dynamics at intermediate dispersal rates. This destabilization is stronger when the ratio between the predator and the prey dispersal rates is higher (Fig. S3 *versus* Fig. S4).

At high dispersal rates, the metacommunity is a well-mixed system, i.e. the population dynamics are identical in all patches. When regional enrichment is above the threshold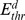, the equilibrium becomes unstable and prey and predator population dynamics fluctuate over time (Fig. 2B, C). The threshold value of regional enrichment 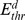 (black line, Fig. 3, Fig. S1) tends to the threshold value of the well-mixed metacommunity 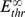 despite differences between prey and predator dispersal rates (Fig. S3, Fig. S4). In the enriched patch, the amplitude of prey and predator dynamics increases with regional enrichment, but remains lower than at low dispersal rate (Fig. S1). Similarly, the minimal densities decrease with increasing regional enrichment, but remain higher than at low dispersal rate (Fig. 3). By contrast, in the poor patch the amplitude of the dynamics increases with increasing regional enrichment beyond the values at low dispersal rate (Fig. S1). In the same way, the minimal densities in the enriched patch decrease with increasing regional enrichment and are lower than those reached at low dispersal rates (Fig. 3). Thus when regional enrichment is high and is distributed heterogeneously, high dispersal rates stabilize population dynamics in the enriched patch at the expense of population stability in the other patch. This result is robust to differences between prey and predator dispersal rates (Fig. S3, Fig. S4).

## Discussion

Using a spatial Rosenzweig-MacArthur model where communities are linked by passive and global dispersal, we found that the spatial distribution of enrichment and the dispersal of organisms between patches modify the response of predator-prey metacommunities to enrichment. More precisely we found that when the enrichment is even among communities, it destabilizes the metacommunity regardless of the dispersal. In contrast, we found that when some communities are more enriched than others, dispersal can stabilize the metacommunity. The strength of this stabilizing effect depends on the number of patches and on the resource availability for the prey (carrying capacity) in the metacommunity before enrichment. Thus our results show that resource enrichment does not necessarily lead to instability in metacommunities if the enrichment is heterogeneous and the communities highly connected. Hence, the spatial heterogeneity of enrichment accompanied by high dispersal rates can prevent or dampen the paradox of enrichment, and more generally, the destabilizing effect of energy flux on predator-prey dynamics [3].

The stabilizing effect of dispersal we have found here is different from the “statistical stabilization” mechanism described previously by Jansen [18] and others [27], [28], [38], [46]. The statistical stabilization arises when predator-prey dynamics are spatially asynchronous at intermediate dispersal rates. The amplitude of regional densities of the prey and of the predator is then dampened despite strong fluctuations of population densities at the local scale. By contrast, the stabilizing effect of dispersal at work here occurs at high dispersal rates when prey and predator densities are homogenized and hence when population dynamics are spatially synchronous. This stabilizing effect of dispersal without spatial asynchrony is related to a second mechanism, the “non-linear spatial averaging”, described by Briggs and Hoopes [27]. These authors describe how spatial predator-prey dynamics can lead to heterogeneous distribution of prey density whereas predator density remains uniform in space (e.g. [37], [47]). Predators foraging in such heterogeneous landscape will have their conversion efficiency reduced by comparison to uniform landscape because of the non-linear predator-prey interaction (functional response type II) [37], [47]. This reduction in the conversion efficiency leads then to the stabilization of the predator-prey dynamics [48]. In our model, the mechanism is similar but acts at the prey level, which has a non-linear intrinsic growth rate (logistic growth). The spatial heterogeneity of enrichment leads to the variation of the resource for the prey from patch to patch (carrying capacity). At high dispersal rates, the prey, whose density is uniform in the landscape, experiences a regional resource availability (harmonic mean) that is lower when the local carrying capacities are heterogeneous than when they are uniform. This decrease of regional resources availability in heterogeneous landscapes decreases the energy flux between the prey and its predator, which leads to the stabilization of the population dynamics in our model. Finally, the spatial heterogeneity in resources (or in other parameters) can simultaneously favor a third mechanism of stabilization by dispersal [49], [50]. This third mechanism arises when the stability is improved by the uncoupling of immigration from local dynamics [27], [35]. The spatial heterogeneity in resource (or in other parameters) causes the local dynamics to be asynchronous in patches when in isolation. Then dispersal between patches can lead to negative density-dependence in the per capita growth rate which stabilizes predator-prey dynamics [27]. This third mechanism is also probably at work in the stabilizing effect of dispersal observed in our results. To conclude, our results illustrate that the principle of energy flux can be dampened by dispersal in spatially heterogeneous landscape through the “non-linear averaging” and the “uncoupling of immigration from local dynamics” mechanisms. Similar results were found in models where adaptive movements of the top predator (i.e. movements that depend on patch quality or local densities) connect local food webs [51]–[53]. In such models, the spatial heterogeneity in consumption rates or other parameters stabilizes the population dynamics [51]–[53].

We explored a wide range of dispersal rates, which corresponds to a large range of spatial scales. Indeed, for a given prey and its predator, the increase of dispersal rates means that the residence time of individuals in patches after between-patch movement decreases with respect to their generation time. The higher the dispersal rates, the lower individuals spend time in a given patch after their arrival in this patch. Hence, low dispersal rates represent ecological situation where the scale of movements of organisms is smaller than the scale of spatial heterogeneity, whereas high dispersal rates correspond to the inverse situation. At high dispersal rates in our model, movements of organisms between patches are frequent, such as the foraging movements of a predator searching for resources in various habitat patches. The differences in the carrying capacity between patches represent spatial heterogeneity at smaller spatial scale than the scale of organisms' movements.

Because the stabilizing mechanism underlined here requires high dispersal rates, it could be at work in communities where the distribution of the resource for the prey is heterogeneous at small spatial scale with respect to the scale of organisms' movements. Within communities, prey and their predators frequently move between habitat patches of varying resource availabilities. For instance, lakes are structured by a vertical gradient of light from the surface to deep water leading to heterogeneous distribution of primary producers in the water column [24]. As a consequence, resource availability for zooplankton, which move frequently between the pelagic part and the benthic part beneath as their predators (fishes), is spatially heterogeneous [25]. In such fish-zooplankton community, the spatial heterogeneity of resource availability for zooplankton might lead to a higher stability threshold and hence to a higher robustness of the community to enrichment destabilization. If this spatial heterogeneity in prey resource is not taken into account in such heterogeneous community, we may overestimate the potential for variability in predator-prey dynamics. This will also be the case when the spatial distribution of enrichment is heterogeneous which increases spatial heterogeneity in prey resource. For instance, lake eutrophication can increase phytoplankton biomass in upper layers, whereas phytoplankton biomass in deeper layers can be decreased by lower light availability [41]. This increases the spatial heterogeneity of resources (phytoplankton) for the zooplankton. Thus the spatial heterogeneity in resource availability within communities could explain that the paradox of enrichment has not been observed many times in nature (e.g. [54]) and in non-spatial experiments [55].

Our results show also that heterogeneity of resource distribution at small scale with respect to the scale of organisms' movements should decrease the variability of prey and predator population dynamics. In complex terrestrial landscapes where herbivorous insects and their predators move frequently between patches, the abundance of plants consumed by herbivorous insects can vary from patch to patch leading to heterogeneous distribution of the resource of the herbivore. Although empirical studies relating population dynamics to landscape heterogeneity are scarce, and mainly restricted to single species [56], [57], some empirical patterns are consistent with our result. For instance, the variability of population dynamics of several butterflies is negatively correlated with habitat heterogeneity around sampling sites [56]. These habitats differ with respect to a number of variables including the abundance of plants providing resources to butterflies. Habitat heterogeneity could stabilize population dynamics through the mechanism underlined here if the variability of the population dynamics of these butterflies is mainly caused by their interaction with their respective predators. Studies of pest control by natural enemies in agro-ecosystems have also provided insights on the effect of habitat heterogeneity on predator-prey interactions, but data allowing measurement of the stability of population dynamics are rare [26], [58], [59]. For instance, the soybean aphid has exhibited apparently cyclic outbreaks, with high populations in one year, typically followed by low populations in the following year [59]. The biological control of this pest is also positively related to the diversity of habitat (crops, forests, grassland) around sampling sites [59]. Although this study does not relate the heterogeneity of prey resources to population stability explicitly, the stabilizing mechanism underlined here could explain these empirical results.

We found that when dispersal is low the metacommunity equilibrium becomes unstable for lower level of enrichment when the spatial distribution of enrichment is heterogeneous than when it is uniform. When heterogeneous enrichment leads to equilibrium destabilization, prey and predator densities fluctuate strongly in the most enriched patch, which increases the local extinction risk (low minimal densities). By contrast, in the poor patches, population densities remain close to constant and the local extinction risk is very low, even if regional enrichment is high. Such metapopulations, comprising at least one population with stable dynamics and unstable populations prone to local extinction, are considered to have a very low regional extinction risk [20], [60], [61]. Indeed, dispersal from the stable population (here in the less enriched patch) can provide a permanent source for recolonization of patches where local extinctions occurred (here the most enriched patch). Thus, we argue that heterogeneous distribution of enrichment should reduce the risk of regional extinction in the metacommunity. At low dispersal rates and hence at large spatial scale with respect of the scale of prey and predator movement, the risk of regional extinction in metacommunities where prey resource distribution is heterogeneous should remain very low, despite that the metacommunity equilibrium is destabilized.

### Implications for biological control and conservation

Our results found with a simple spatial model show that variation of the spatial scale of resource heterogeneity with respect of the spatial scale of organisms' movements can affect the stability of a predator-prey system.

From the perspective of the conservation of species involved in specialized predator-prey interaction, our results suggest a simple qualitative pattern: resource heterogeneity for prey species promotes low regional extinction risk of the prey and of the predator. When resource heterogeneity occurs at large spatial scale (low dispersal rates), the presence of patches poor in prey resource provides stable local dynamics that may ultimately allow the recolonization of patches where high resource availability and predator-prey interaction have led to local extinctions. As a consequence, the spatial heterogeneity improves the regional persistence of the prey and its predator. Moreover, when resource heterogeneity occurs at small spatial scale (high dispersal rates), we found that dispersal can stabilize the population dynamics of the prey and of its predator in the whole metacommunity. Thus our results suggest that improving spatial heterogeneity at small and at large spatial scale should favor low regional extinction risk of species involved in specialized predator-prey interaction and hence should promote species persistence. This prediction is relevant for species that disperse passively from patches to patches. Indeed, when predator dispersal is adaptive (i.e. depends on patch quality), spatial heterogeneity of resources for the prey can rather lead to population destabilization [52].

The qualitative implications of our results for the biological control of pests by specialized natural enemies are more complicated. In agricultural landscapes made of patches with crops and with semi-natural patches such as grassland, crop patches provide habitat with high resource availability (carrying capacity) for herbivorous pests whereas the resource availability in semi-natural patches is lower. When landscape structure provides spatial heterogeneity of prey resources at a large spatial scale with respect to the spatial scale of movements (low dispersal rates), our model suggests that crop patches should be damaged by pest outbreaks whereas semi-natural patches will provide a permanent source of pest. In this case, spatial heterogeneity might be detrimental to crop yield. However, if the spatial heterogeneity of the pest's resources is increased at a small spatial scale (high dispersal rates), our results show that frequent movements of the pest and of specialized natural enemies between these two types of patch may decrease the amplitude of population dynamics and hence prevent outbreaks responsible for detrimental crops damage. Thus our results suggest that the improvement of heterogeneity of pest resources at a small spatial scale (with respect to the scale of movements of pests and of their natural enemies) could decrease the risk of pest outbreaks. This effect of the spatial scale of resource heterogeneity for the pest, shown in our simple model, might provide a trail for the understanding of the observed variability in the relationship between landscape heterogeneity and the risk of pest outbreaks in the field [59], [62].

### Caveats and future work

Our model has considered passive dispersal of the prey and of the predator, but other dispersal rules are possible. For instance, species dispersal can be density-dependent [63] or fitness-dependent, Such dispersal rules can affect the stability of predator-prey dynamics [45] and species coexistence within trophic modules [64], [65]. Moreover, we considered global dispersal (the dispersal rate is the same between all pairs of patch). These simple hypotheses allowed us to perform analytical study of the effect of enrichment when dispersal is high. Our results show that spatial heterogeneity of enrichment can promote the stability of population dynamics at high dispersal rates regardless to the patch number and the model parameterization. So this study is a first step and it would be interesting to investigate more complex spatial structures of enrichment heterogeneity in landscapes where prey and predator dispersal is localized (e.g. dispersal to the closest neighbor), which is possible in spatially explicit models. The metacommunity theory is general enough to incorporate these additional complexities, and future directions should address these issues and other important questions, such as the role of nutrient recycling within metaecosystems, which can have important consequences on the dynamics of prey and their predators [22].

## Supporting Information

Appendix S1 **Analysis of metacommunities dynamics when dispersal tends to zero or infinity**
(PDF)

Figure S1
**Effect of regional enrichment (** ***E*** **) and of dispersal rate (** ***d_N_*** **=** ***d_P_*** **) on the amplitude of population dynamics when spatial heterogeneity is maximal (α = 1).** When enrichment is zero, the carrying capacities are equal to *K*_0_ and they are below the stability threshold of an isolated patch. Regional enrichment (*E*) increases the carrying capacity in the enriched patch (*K_0_*+*E)*, whereas the carrying capacity in the poor patch is kept constant (*K_0_*). The equilibrium of the two-patch metacommunity (*M* = 2) is stable below the black line. The amplitude of density fluctuation of the predator (A,B) and of the prey (C,D) in the poor patch (A,C) and in the enriched patch (B,D) is represented with grey levels. The higher is the amplitude, the lower the stability of population dynamics. At the three points labeled A, B and C, the population dynamics are illustrated in Figure 2. The solid lines then represent the enrichment threshold, 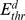, for which the metacommunity is destabilized. 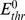
and 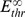 denote the regional enrichment thresholds found analytically respectively for an isolated patch (*d_N_* = *d_P_*  = 0) and for the well-mixed metacommunity (*d_N_* = *d_P_*  = +∞). Parameters values: *r* = 10, *K_0_* = 18.3, *e* = 0.1, *a* = 5, *t_h_* = 0.01, *m* = 1.(TIFF)

Figure S2
**Sensitivity of the effect of heterogeneous regional enrichment (α = 1) on the stability threshold of the equilibrium** (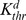) **to changes in prey and predator parameters.** Metacommunities have two-patches (*M* = 2), one poor patch where the prey carrying capacity (*K_1_*) is kept constant, and one enriched patch where prey carrying capacity (*K_2_* =  *K_1_*+*E*) is increased. Δ is defined as the relative difference between the carrying capacity in the enriched patch for which the equilibrium of the metacommunity is stable (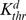) and the maximal carrying capacity for which the equilibrium is stable when patches are isolated (*K*_thr_). We explored two values of *K*_1_. For each of the 100 dispersal rate values and for each of the two *K*_1_ values, we performed 100 replicates with parameter values taken randomly within the following ranges: *r* = [1 10], *e* = [0.1,1], *a* = [1], [10], *t_h_* = [0.01,0.1], *m* = [0.1,1], *d_P_* = *d_N_*. This figure shows that parameters changes do not affect qualitatively the relationship between dispersal rates and the stability threshold at intermediate dispersal rates.(TIFF)

Figure S3 **Effect of regional enrichment (** ***E*** **) and of dispersal rate on the amplitude of population dynamics when spatial heterogeneity is maximal (α = 1) and when the dispersal rate of the prey (** ***d*** **_N_) is lower than the dispersal rate of the predator (** ***d*** **_P_).** See legend of fig. S1 for explanations and parameter values except *d*_P_  =  100 *d_N_*.
(TIFF)

Figure S4 **Effect of regional enrichment (** ***E*** **) and of dispersal rate on the amplitude of population dynamics when spatial heterogeneity is maximal (α = 1) and when the dispersal rate of the prey (** ***d*** **_N_) is higher than the dispersal rate of the predator (** ***d*** **_P_).** See legend of fig. S1 for explanations and parameter values except *d*_P_ = 0.01 *d_N_*.
(TIFF)
