# Supplementary material for: Confronting the Paradox of Enrichment to the Metacommunity Perspective
Source: PLoS One. 2013 Dec 16;8(12):e82969. doi: 10.1371/journal.pone.0082969 (PMC3865114; doi:10.1371/journal.pone.0082969)
Supplement: Appendix S1 — Analysis of metacommunities dynamics when dispersal tends to zero or infinity (PDF) [file pone.0082969.s001.pdf]

APPENDIX S1

ANALYSIS OF METACOMMUNITIES DYNAMICS WHEN DISPERSAL TENDS TO ZERO OR INFINITY

Confronting the paradox of enrichment to the metacommunity perspective

Céline Hauzy * [1,2,3], Grégoire Nadin [4], Elsa Canard [5], Isabelle Gounand [5], Nicolas Mouquet [5] & Bo Ebenman [1]

1. Department of Physics, Chemistry and Biology, Linköping University, Linköping, Sweden
2. Université Pierre et Marie Curie, UMR7625 - Ecologie et Evolution, Paris, France
3. Institut National de la Recherche Agronomique, USC2031 - Ecologie des Populations et Communautés, Paris, France
4. CNRS, UMR7598 - Laboratoire Jacques-Louis Lions, Paris, France
5. Institut des Sciences de l'Evolution, Université de Montpellier II, Montpellier, France.

* Corresponding author: celinehauzy@gmail.com

A. DYNAMICS OF METACOMMUNITIES WHEN DISPERSAL TENDS TO INFINITY

We consider a predator-prey metacommunity occupying *M* patches where prey growth follows a logistic shape, with any prey-dependent functional response *f* and predator mortality *g*. *D*ispersal depends on density in the departure patch.

$$\begin{cases} \dfrac{dN_i}{dt} = r\,N_i\left(1-\dfrac{N_i}{K_i}\right) - f(N_i)P_i - d_N N_i + \dfrac{1}{M-1}\sum_{\substack{j=1\\ j\neq i}}^{M} d_N N_j \\ \dfrac{dP_i}{dt} = e\,f(N_i)P_i - g(P_i)P_i - d_P P_i + \dfrac{1}{M-1}\sum_{\substack{j=1\\ j\neq i}}^{M} d_P P_j \end{cases} \quad \text{(A.1)}$$

Our aim is to investigate the properties of the solutions when d_N and $d_P \to +\infty$. In order to facilitate the analysis, we assume $d_N = d_P = d$. For any *d,* we denote (N_i^d , P_i^d) the associated solution of (A.1).

First, the equation A.1 of the prey for each patch *i* is equivalent to:

$$\frac{1}{d}\left(-\frac{dN_i^d}{dt} + rN_i^d\left(1-\frac{N_i^d}{K_i}\right) - f(N_i^d)P_i^d\right) = N_i^d - \frac{1}{M-1}\sum_{\substack{j=1\\ j\neq i}}^{M} N_j^d \quad \text{(A.2)}$$

When $d \to +\infty$, the left-hand side of (A.2) tends to 0 and thus $N_i^d \to N_i^\infty$, with

$N_i^\infty = \dfrac{1}{M-1}\sum_{\substack{j=1\\ j\neq i}}^{M} N_j^\infty$. This implies that N_i^∞ is independent of *i*. The same conclusion is obtained for the equation of the predator. This means that the prey and the predator have the same densities in all patches when dispersal is global and infinite. Set N^∞ and P^∞ these densities.

Second, we want to find the equations describing the densities (N^∞, P^∞) in the metacommunity when dispersal tends to infinity. Notice that $\sum_{i=1}^{M} N_i^d = \frac{1}{M-1}\sum_{i=1}^{M}\sum_{\substack{j=1\\ j\neq i}}^{M} N_j^d$ and $\sum_{i=1}^{M} P_i^d = \frac{1}{M-1}\sum_{i=1}^{M}\sum_{\substack{j=1\\ j\neq i}}^{M} P_j^d$. Thus, taking the sums of equations (A.1) over *i* leads to:

$$\begin{cases} \sum_{i=1}^{M} \frac{dN_i^d}{dt} = \sum_{i=1}^{M}\left[r\,N_i^d\left(1-\frac{N_i^d}{K_i}\right) - f(N_i^d)P_i^d \right] \\ \sum_{i=1}^{M} \frac{dP_i^d}{dt} = \sum_{i=1}^{M}\left[e\,f(N_i^d)P_i^d - g(P_i^d)P_i^d \right] \end{cases} \qquad \text{(A.3)}$$

When $d \to +\infty$, $N_i^d \to N^\infty$ and $P_i^d \to P^\infty$, and the system of equation (A.3) tends to:

$$\begin{cases} \frac{dN^\infty}{dt} = rN^\infty\left(1-\frac{N^\infty}{\overline{K}}\right) - f(N^\infty)P^\infty \\ \frac{dP^\infty}{dt} = e\,f(N^\infty)P^\infty - g(P^\infty)P^\infty \end{cases} \qquad \text{(A.4)}$$

where $\overline{K} = M\left(\sum_{i=1}^{M}\frac{1}{K_i}\right)^{-1}$ is the harmonic mean of the carrying capacities K_i in the different patches *i*.

Third, we conclude on the stability of the equilibrium for our specific model. If the functional response is of type II (Holling 1959), $f(N^\infty) = \frac{aN^\infty}{1+at_hN^\infty}$, and if *g* is a constant $g(P^\infty) = m$, we obtained the Rosenzweig-MacArthur model. This model has one equilibrium where the prey and the predator can coexist. At this equilibrium, predator density is positive when $\overline{K} > \frac{m}{a(e-mt_h)}$. By studying the sign of the determinant and of the trace of the Jacobian matrice of (A.4), it is easy to show that this equilibrium is stable when $\overline{K} < K_{thr}$ with $K_{thr} = \frac{e+mt_h}{at_h(e-mt_h)}$.

B. EFFECTS OF REGIONAL ENRICHMENT ON METACOMMUNITIES

B.1 Stability thresholds

We consider a predator-prey metacommunity occupying *M* patches where the carrying capacity in all the patches is assumed to be K_0, which is below the destabilizing threshold of an isolated patch K_{thr}. We consider that this metacommunity experiences regional enrichment *E* and we studied the critical value of regional enrichment, E_{thr}, leading to the destabilization of metacommunity equilibrium in the limiting cases where there is no dispersal and where dispersal tends to infinity.

(a) No dispersal

As we consider that dispersal from patch to patch is equal to zero, the metacommunity equilibrium is stable if the equilibriums of each of the *M* isolated communities are stable. Isolated communities have one equilibrium where the prey and the predator can coexist with positive densities if $K_i > m/(a(e-mt_h))$. This equilibrium is stable if and only if the carrying capacity K_i in this patch is lower than $K_{thr} = (e+mt_h)/(at_h(e-mt_h))$. If the carrying capacity of at least one community crosses the threshold K_{thr}, then metacommunity equilibrium is unstable.

When enrichment distribution is homogeneous (α=0), the carrying capacity in each patch is equal to K_0+E/M. Thus, the equilibrium of the metacommunity is destabilized when the regional enrichment *E* exceeds the critical value $E^0_{thr}(\alpha=0)=(K_{thr}-K_0)M$.

When the spatial heterogeneity in enrichment distribution is maximal (α=1), the regional enrichment *E* is concentrated in only one patch. When enrichment leads the carrying capacity in this patch to be higher than K_{thr}, the equilibrium of the metacommunity is destabilized. Thus, the equilibrium of the metacommunity is destabilized when enrichment is higher than the critical value $E_{thr}^{0}(\alpha=1)=(K_{thr}-K_0)$.

(b) Infinite dispersal

We consider that dispersal from patch to patch tends to +∞. The dynamics of this homogenized metacommunity are described by the equations (A.4).

When enrichment distribution is homogeneous (α=0), the carrying capacity in each patch is increased by the same amount *E*/*M*. Hence the carrying capacity is the same in all patches and is equal to K_0+*E*/*M*. As a consequence, the regional carrying capacity of the prey simplify as: $\overline{K}=K_i=K_0+E/M$. Thus, the equilibrium of the metacommunity is destabilized when enrichment is higher than the critical value $E_{thr}^{\infty}(\alpha=0)=(K_{thr}-K_0)M$.

When the heterogeneity of enrichment distribution is maximal (α=1), only one patch receives enrichment and its carrying capacity is increased to K_0+*E* whereas the carrying capacity in the other patches is equal to K_0, Hence, the regional carrying capacity of the prey is:

$$\overline{K}=M\left(\sum_{i=1}^{M}\frac{1}{K_i}\right)^{-1}=M\left(\frac{1}{K_0+E}+\sum_{i=1}^{M-1}\frac{1}{K_0}\right)^{-1}=M\left(\frac{1}{K_0+E}+\frac{M-1}{K_0}\right)^{-1} \quad \text{(B.1)}$$

If the regional enrichment *E* tends to +∞ in (B.1), then $\overline{K}$ tends to a maximal value $\overline{K}_{\max}$:

$$\overline{K}\xrightarrow{E\to\infty}\frac{M}{M-1}K_0=\overline{K}_{\max}$$

Recall that the equilibrium of the homogenized metacommunity is stable if $\overline{K} < K_{thr}$ where K_{thr} depends only on predator parameters. Hence, there are two cases:

- In the first case, where $K_0 \times M/(M-1) < K_{thr}$, the regional enrichment E does never destabilize the equilibrium of the homogenized metacommunity, even the regional enrichment reaches infinite values.

  In the second case, where $K_0 \times M/(M-1) > K_{thr}$, the regional enrichment E destabilizes the equilibrium of the homogenized metacommunity when it is above the threshold given by:

$$E_{thr}^{\infty}(\alpha=1) = \left(\frac{M}{K_{thr}} - \frac{M-1}{K_0}\right)^{-1} - K_0 = \frac{K_0 \times M(K_{thr} - K_0)}{M K_0 - (M-1)K_{thr}}.$$

  We found that $\frac{\partial E_{thr}^{\infty}(\alpha=1)}{\partial M} > 0$ if $K_{thr} > K_0$, $\frac{\partial E_{thr}^{\infty}(\alpha=1)}{\partial K_0} < 0$ and $\frac{\partial E_{thr}^{\infty}(\alpha=1)}{\partial K_{thr}} > 0$.

B.2 Comparison of stability thresholds

Now, we want to compare these four thresholds in order to study the effect of enrichment distribution and of dispersal on the stability of metacommunity equilibrium.

(a) Effect of enrichment distribution when dispersal is null: $E_{thr}^{0}(\alpha=0)$ *versus* $E_{thr}^{0}(\alpha=1)$.

We have $E_{thr}^{0}(\alpha=0) = (K_{thr} - K_0)M \quad > \quad (K_{thr} - K_0) = E_{thr}^{0}(\alpha=1)$. Thus, when dispersal is null, the equilibrium of the metacommunity is destabilized at higher level of regional enrichment when the distribution of enrichment is homogeneous (α=0) than when it is heterogeneous (α=1).

(b) Effect of enrichment distribution when dispersal tends to infinity: $E_{thr}^{\infty}(\alpha=0)$ *versus* $E_{thr}^{\infty}(\alpha=1)$.

Because we assume that before enrichment the carrying capacities in all patches are below the destabilization threshold of an isolated patch ($K_0 < K_{thr}$), we can compute:

$$E_{thr}^{\infty}(\alpha=1)=\frac{K_0\times M(K_{thr}-K_0)}{MK_0-(M-1)K_{thr}} \quad > \quad \frac{K_0\times M(K_{thr}-K_0)}{MK_0-(M-1)K_0}=M(K_{thr}-K_0)=E_{thr}^{\infty}(\alpha=0)$$

Thus, when dispersal is infinite, the equilibrium of the homogenized metacommunity is destabilized for lower levels of regional enrichment when enrichment distribution is homogeneous than when the heterogeneity of enrichment distribution is maximal.

(c) Effect of dispersal when α*=0:* $E_{thr}^{0}(\alpha=0)$ *versus* $E_{thr}^{\infty}(\alpha=0)$.

We have $E_{thr}^{0}(\alpha=0)=(K_{thr}-K_0)M=E_{thr}^{\infty}(\alpha=0)$. Thus, when enrichment distribution is homogeneous, the equilibrium of the metacommunity is destabilized for the same level of regional enrichment when dispersal is null and when dispersal is infinite.

(d) Effect of dispersal when α*=1:* $E_{thr}^{0}(\alpha=1)$ *versus* $E_{thr}^{\infty}(\alpha=1)$.

Because we assume $K_0 < K_{thr}$, we can compute:

$$E_{thr}^{\infty}(\alpha=1)=\left(\frac{M}{K_{thr}}-\frac{M-1}{K_0}\right)^{-1}-K_0 \quad > \quad \left(\frac{M}{K_{thr}}-\frac{M-1}{K_{thr}}\right)^{-1}-K_0=K_{thr}-K_0=E_{thr}^{0}(\alpha=0)$$

Thus, if the heterogeneity of enrichment distribution is maximal, the destabilization of the metacommunity equilibrium occurs for lower levels of regional enrichment when dispersal is null than when dispersal tends to infinity.
