# Supplementary figures and images for: Confronting the Paradox of Enrichment to the Metacommunity Perspective
Source: PLoS One. 2013 Dec 16;8(12):e82969. doi: 10.1371/journal.pone.0082969 (PMC3865114; doi:10.1371/journal.pone.0082969)

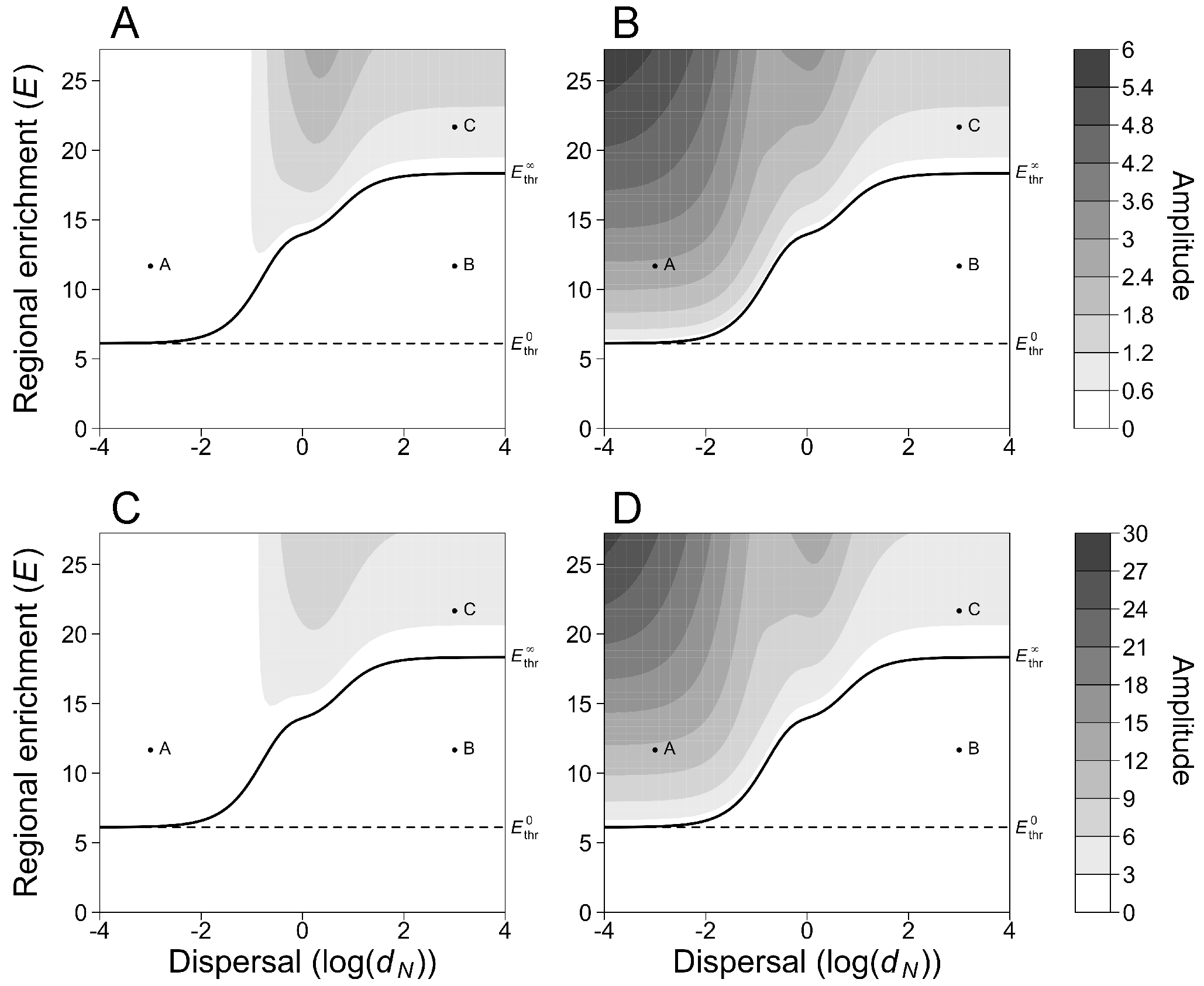

Supplement: Figure S1 — Effect of regional enrichment ( E ) and of dispersal rate ( dN = dP ) on the amplitude of population dynamics when spatial heterogeneity is maximal (α = 1). When enrichment is zero, the carrying capacities are equal to K 0 and they are below the stability threshold of an isolated patch. Regional enrichment (E) increases the carrying capacity in the enriched patch (K0+E), whereas the carrying capacity in the poor patch is kept constant (K0). The equilibrium of the two-patch metacommunity (M = 2) is stable below the black line. The amplitude of density fluctuation of the predator (A,B) and of the prey (C,D) in the poor patch (A,C) and in the enriched patch (B,D) is represented with grey levels. The higher is the amplitude, the lower the stability of population dynamics. At the three points labeled A, B and C, the population dynamics are illustrated in Figure 2. The solid lines then represent the enrichment threshold, , for which the metacommunity is destabilized. and denote the regional enrichment thresholds found analytically respectively for an isolated patch (dN = dP = 0) and for the well-mixed metacommunity (dN = dP = +∞). Parameters values: r = 10, K0 = 18.3, e = 0.1, a = 5, th = 0.01, m = 1. (TIFF) [file pone.0082969.s002.tiff]

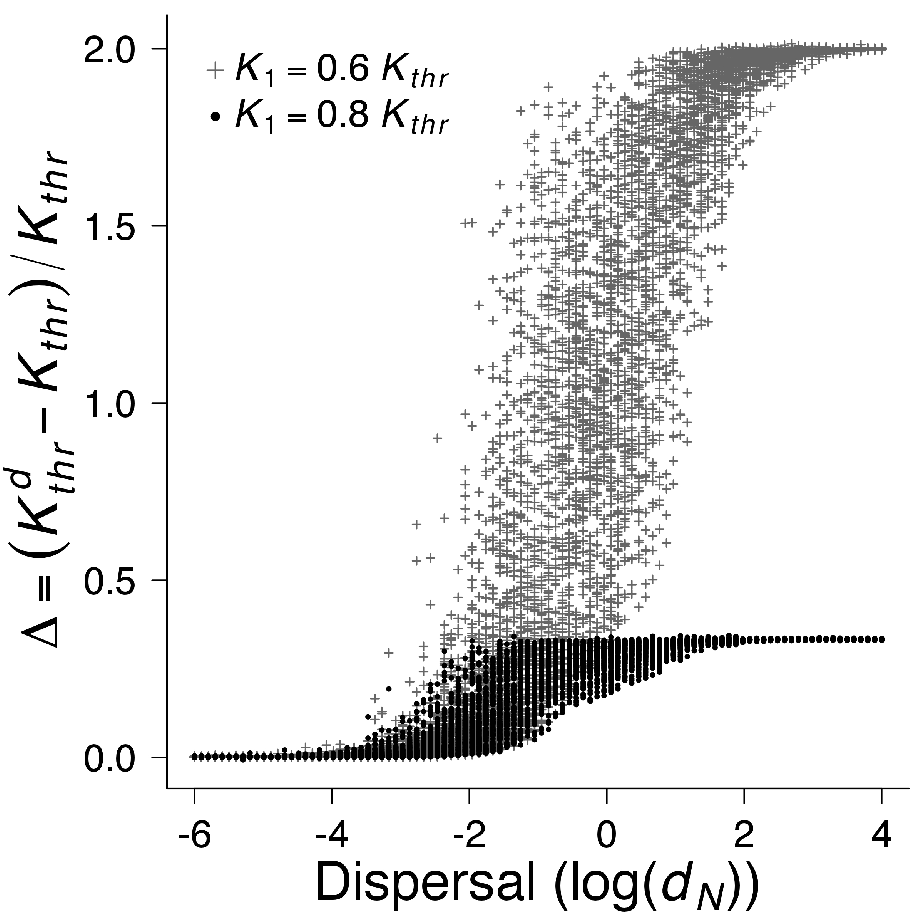

Supplement: Figure S2 — Sensitivity of the effect of heterogeneous regional enrichment (α = 1) on the stability threshold of the equilibrium () to changes in prey and predator parameters. Metacommunities have two-patches (M = 2), one poor patch where the prey carrying capacity (K1) is kept constant, and one enriched patch where prey carrying capacity (K2 = K1+E) is increased. Δ is defined as the relative difference between the carrying capacity in the enriched patch for which the equilibrium of the metacommunity is stable () and the maximal carrying capacity for which the equilibrium is stable when patches are isolated (K thr). We explored two values of K 1. For each of the 100 dispersal rate values and for each of the two K 1 values, we performed 100 replicates with parameter values taken randomly within the following ranges: r = [1 10], e = [0.1,1], a = [1], [10], th = [0.01,0.1], m = [0.1,1], dP = dN. This figure shows that parameters changes do not affect qualitatively the relationship between dispersal rates and the stability threshold at intermediate dispersal rates. (TIFF) [file pone.0082969.s003.tiff]

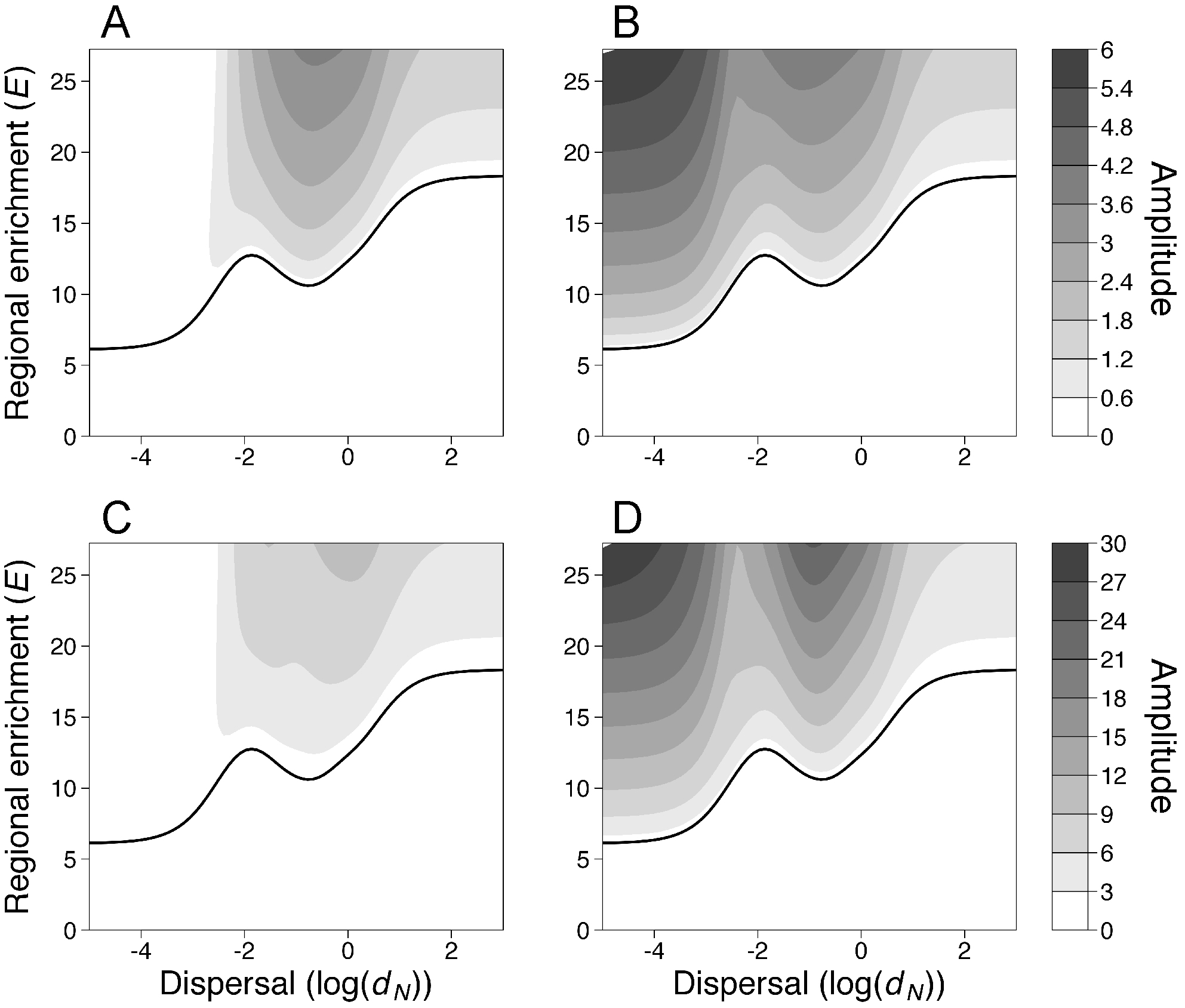

Supplement: Figure S3 — Effect of regional enrichment ( E ) and of dispersal rate on the amplitude of population dynamics when spatial heterogeneity is maximal (α = 1) and when the dispersal rate of the prey ( d N) is lower than the dispersal rate of the predator ( d P). See legend of fig. S1 for explanations and parameter values except d P = 100 dN. (TIFF) [file pone.0082969.s004.tiff]

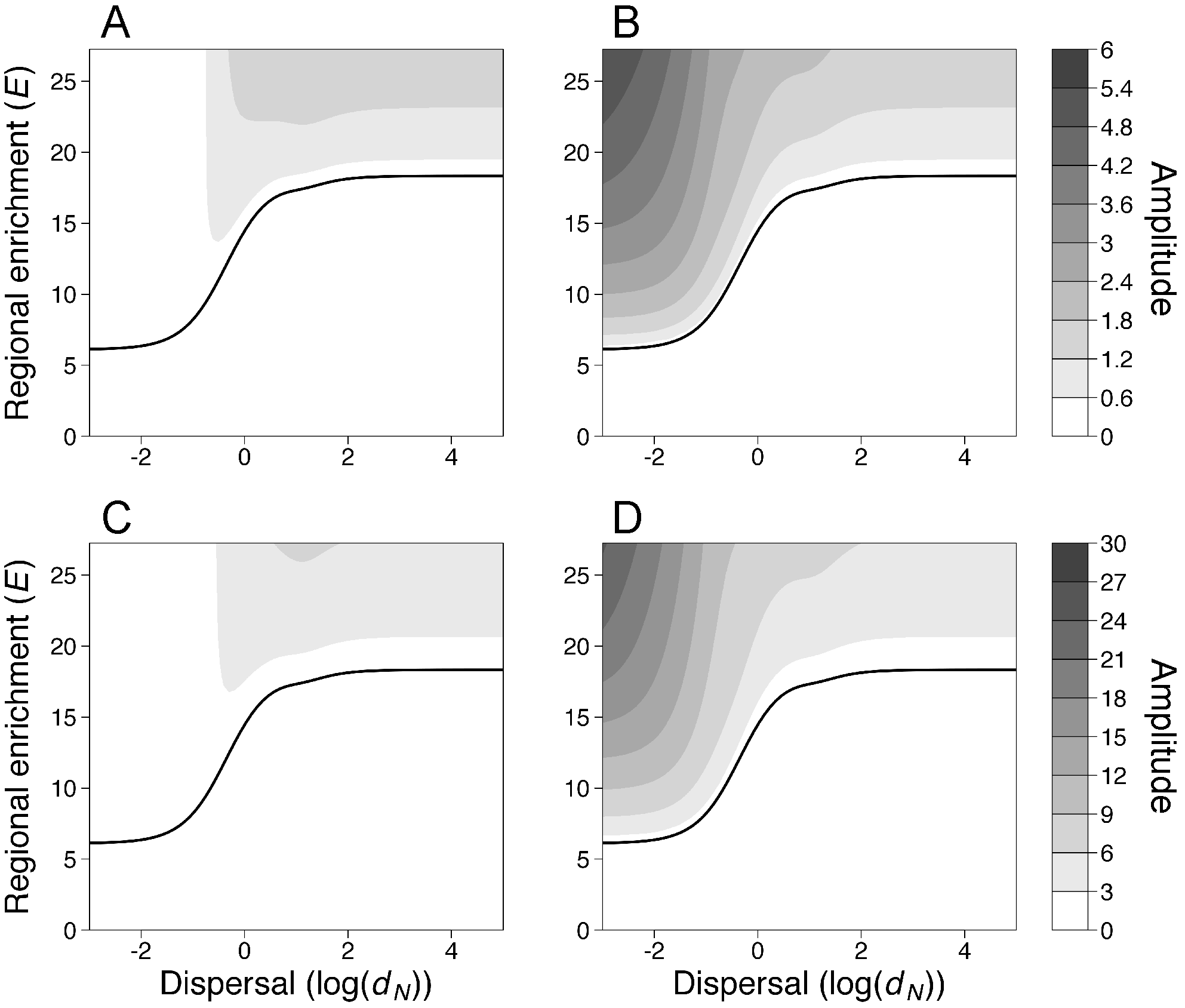

Supplement: Figure S4 — Effect of regional enrichment ( E ) and of dispersal rate on the amplitude of population dynamics when spatial heterogeneity is maximal (α = 1) and when the dispersal rate of the prey ( d N) is higher than the dispersal rate of the predator ( d P). See legend of fig. S1 for explanations and parameter values except d P = 0.01 dN. (TIFF) [file pone.0082969.s005.tiff]
